# Assessment of aggressive bladder cancer mutations in plasma cell-free DNA

**DOI:** 10.3389/fonc.2023.1270962

**Published:** 2023-11-30

**Authors:** Raquel Carrasco, Mercedes Ingelmo-Torres, Josep Oriola, Fiorella L. Roldán, Leonardo Rodríguez-Carunchio, Sandra Herranz, Begoña Mellado, Antonio Alcaraz, Laura Izquierdo, Lourdes Mengual

**Affiliations:** ^1^ Laboratori i Servei d’Urologia, Hospital Clínic de Barcelona, Barcelona, Spain; ^2^ Genètica i tumors urològics, Fundació de Recerca Clínic Barcelona-Institut d’Investigacions Biomèdiques August Pi i Sunyer (FRCB-IDIBAPS), Barcelona, Spain; ^3^ Departament de Biomedicina, Facultat de Medicina i Ciències de la Salut, Universitat de Barcelona (UB), Barcelona, Spain; ^4^ Servei d’Anatomia Patològica, Hospital Clínic de Barcelona, Barcelona, Spain; ^5^ Servei d’Oncologia Mèdica, Hospital Clínic de Barcelona, Barcelona, Spain

**Keywords:** bladder cancer, cell-free DNA, metastasis, mutation, tumor heterogeneity

## Abstract

**Background and aims:**

The spatial and temporal genetic heterogeneity of bladder cancer (BC) makes challenging to find specific drivers of metastatic disease, thus preventing to determine those BC patients at high risk of tumor progression. Our aim was to identify DNA mutations providing aggressive behavior to bladder tumors and analyze them in patients’ cell-free DNA (cfDNA) during their follow-up after radical cystectomy (RC) in order to monitor tumor evolution.

**Methods:**

Six BC patients who underwent RC and presented disease progression during their follow-up were included. Next-generation sequencing was used to determine somatic mutations in several primary tumor and metastatic specimens from each patient. Shared DNA mutations between primary bladder tumor and metastatic sites were identified in cfDNA samples through droplet digital PCR.

**Results:**

Besides BC genetic heterogeneity, specific mutations in at least one of these genes —*TERT*, *ATM*, *RB1*, and *FGFR3*— were found in primary tumors and their metastases in all patients. These mutations were also identified in the patients’ cfDNA at different follow-up time points. Additionally, the dynamic changes of these mutations in cfDNA allowed us to determine tumor evolution in response to treatment.

**Conclusion:**

The analysis of BC mutations associated with poor prognosis in plasma cfDNA could be a valuable tool to monitor tumor evolution, thus improving the clinical management of BC patients.

## 1 Introduction

Approximately 25% of bladder cancers (BC) patients are diagnosed as muscle-invasive bladder cancer (MIBC). Radical cystectomy (RC) with lymphadenectomy, preceded by neoadjuvant chemotherapy in eligible patients, is the standard treatment for localized MIBC. However, despite undergoing radical surgery, up to 50% of MIBC patients will develop local relapse or distant metastasis within the first two years of RC (1). Metastatic BC patients have a poor prognosis with a five-year relative survival of 6% (2). These patients are generally offered platinum-based chemotherapy; however, the response rate achieved is around 50% (3). An improved understanding of the underlying biology of BC would lead to advances in the treatment and clinical outcomes of this tumor.

The rapid evolution of sequencing technologies has revealed the great complexity of the genomic abnormalities of individual cancer cells and substantial molecular intratumor heterogeneity (ITH), which may evolve over the course of disease and treatment exposure (4). These findings may explain the failure of some approaches toward predictive biomarker development that are exacerbated by tumor sampling bias driven by ITH while also indicating the way forward (5). Examining tumor clonal architecture and its evolution throughout treatment and disease progression may enable the identification of common tumor driver events and individual heterogeneous somatic events present in each tumor that contribute to drug resistance and treatment failure.

Liquid biopsy has emerged to eliminate tumor molecular profile limitations: it is a non-invasive technique, detects tumor clonal evolution in real-time, and reveals ITH (6). Notably, cell-free DNA (cfDNA)-based liquid biopsy has multiple clinical applications and has been studied in different types of tumors, including BC, as a biomarker for detecting molecular relapse or minimal residual disease, monitoring treatment response, or identifying therapy resistance mechanisms (7–11). However, cfDNA-based liquid biopsy has not been implemented in clinical urological practice yet. This is most likely because of the high BC genetic intra- and inter-lesion heterogeneity that makes a challenge to identify those biomarkers predicting which patients are at high risk of metastatic relapse and consequently guiding treatment decisions. In this study, we aimed to determine DNA mutations providing aggressive behavior to bladder tumors and analyze them in patients’ cfDNA during their follow up after RC. The identification of these mutations in bloodstream could be helpful to monitor tumor evolution in response to treatments, thus improving clinical management of bladder cancer patients.

## 2 Materials and methods

### 2.1 Patients and samples

Six BC patients, who underwent RC and lymphadenectomy between 2011 and 2019 at our center and showed disease progression during their follow-up, were included in this study. The exclusion criterion was the presence of another active neoplasm. The clinicopathological features and treatments received by the patients enrolled are summarized in **Supplementary Table S1**. None of the patients received neoadjuvant chemotherapy. Tumor dissemination was controlled postoperatively via a computed tomography scan at three-month intervals during the first year, six-month intervals for the next two years, and annually thereafter. Tumors were considered progressing when relapse or distant metastasis developed during follow-up.

**Figure 1:**
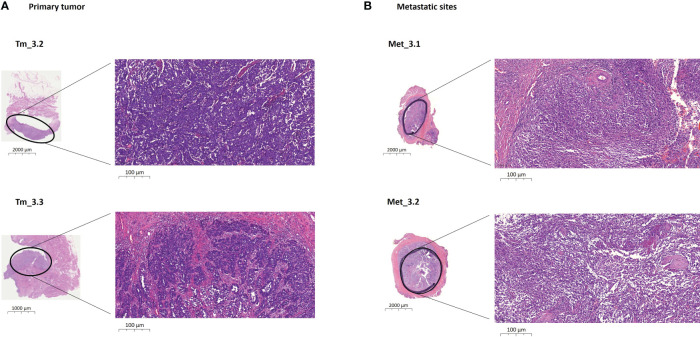
Example of biopsies obtained from a primary bladder tumor and distant metastases from one patient (Pt#3). **(A)** Images of histological regions (Tm_3.2 and Tm_3.3) stained with H&E from the RC specimen at different scales. These regions present a morphology of high-grade conventional urothelial carcinoma that invades the subepithelial connective tissue, developing solid nests and pseudoglandular structures. **(B)** Images of histological regions(Met_3.1 and Met_3.2) stained with H&E from the phallectomy specimen at different scales. These regions show tumoral invasion, presenting a sarcomatoid morphology that affects cavernous bodies. H&E, hematoxylin and eosin; Met, metastatic specimen; Pt, patient; RC, radical cystectomy; Tm, primary tumor specimen.

Primary tumor specimens were obtained from cystectomy (N=5) and transurethral resection of bladder tumor (TURBT) (N=1). Metastatic tissue specimens were obtained from locoregional (N=2) and distant (N=3) metastases from the IDIBAPS biobank, except in one patient (Pt#6), whose metastatic biopsy was not available. One to four biopsies were obtained from primary tumors and locoregional/distant metastases from each patient (**Figure 1**; **Supplementary Figure S1**). Tissue sections were stained using hematoxylin and eosin (H&E) staining to differentiate cellular components, according to standard histopathological laboratory protocol.

An immunohistochemistry (IHC) panel composed of three markers was performed to determine the tumor’s molecular subtype. Formalin-fixed paraffin-embedded (FFPE) tissue samples were cut and stained on BenchMark ULTRA VENTANA (Roche Diagnostics, Tucson, AZ) with CK20 (VENTANA CONFIRM anti-Cytokeratin20, SP33 clone), GATA3 (CELL MARQUE GATA3 L50-823 clone), and CK5 (VENTANA anti-cytokeratin 5/6 D5/16B4 clone) antibodies. Neoplasms expressing intense CK20 and/or GATA3 were classified as luminal subtypes. CK5-positive neoplasms with loss of GATA3 or weak positivity were categorized as basal subtypes.

Genomic DNA was isolated from 26 FFPE sections of 20 µm using the RecoverAll Total Nucleic Acid Isolation kit (Ambion, Inc. Austin, TX, USA), according to the manufacturer’s instructions. Then, DNA was quantified by spectrophotometric analysis at 260 nm (NanoDrop Technologies, Wilmington, DE, USA).

One 10 mL EDTA tube of peripheral blood was collected before RC and at one, four, 12, and 24 months after surgery, except for one patient (Pt#5), from whom only one blood sample was collected seven years after RC. Blood samples were stored at 4°C until processed within the following 24 h.

### 2.2 Next-generation sequencing of primary tissue and metastasis

A total of 15 primary tumor and 11 metastasis biopsies were analyzed by NGS using the Oncomine Bladder Panel (Thermo Fisher Scientific, Massachusetts, USA) with six additional genes. The 31 genes involved in this panel are shown in **Supplementary Table S2**. Ten ng of DNA were used for library preparation according to the manufacturer’s instructions. Sequencing was performed on an Ion S5 System using the Ion 540 Chip (Thermo Fisher Scientific). The mean depth of coverage was 1,104X (range 58-2,000X). NGS data were analyzed with Ion Reporter Software v5.12 (Thermo Fisher Scientific) using an Oncomine Extended (5.18) filter to select for pathogenic variants. The NGS data are available in https://www.ncbi.nlm.nih.gov/clinvar/submitters/509290/.

The clonal evolution of the sequenced tumors was estimated with PHYLOViZ v.2.0. The pathogenic variants identified in each tumor region for each patient were used to develop a phylogenetic tree showing tumor evolution.

### 2.3 Blood sample procedures

Blood samples were centrifuged at 3,500 rpm for 15 min at 4°C to separate plasma, followed by plasma centrifugation at 16,000 x g for 10 min at 4°C to remove any remaining cells. Plasma samples were stored at -80°C until cfDNA extraction.

cfDNA was extracted from 2-5 ml of plasma (depending on availability) using the QIAamp Circulating Nucleic Acid kit (Qiagen, Hilden, Germany), according to the manufacturer’s instructions. For two patients (Pt#1 and Pt#2), cfDNA samples at the time of cystectomy and one month later were missed due to technical failures.

### 2.4 Droplet digital PCR

Tumor mutations shared between primary tumors and their metastatic sites were detected in cfDNA samples by droplet digital PCR (ddPCR) using the QX200 Droplet Digital PCR system (Bio-Rad, Watford, UK), following the manufacturer’s instructions. A mutation is considered that has a potential aggressive behavior when is shared between primary tumors and metastatic sites. For each patient, only mutations identified in their tissue (primary tumor and/or metastasis) were subsequently analyzed in their plasma cfDNA (**Supplementary Table S3**).

Assays to detect and quantify the fractional abundance of point mutations and corresponding wild-type alleles were custom-designed using Primer3 Input (version 4.1.0) (**Supplementary Table S4**). The optimal annealing temperature and limit of detection (LOD) were determined for each assay (**Supplementary Methods**). The mutant allele fraction (MAF) was calculated as the number of droplets positive with the mutant amplicon divided by the total droplets positive with the amplicon (wild-type and mutant). The presence of a mutation was considered detectable if MAF > LOD. Data analysis was carried out using QuantaSoft Analysis Pro Software, version 1.0 (Bio-Rad).

## 3 Results

### 3.1 Clinicopathological features of patients

**Figure 2:**
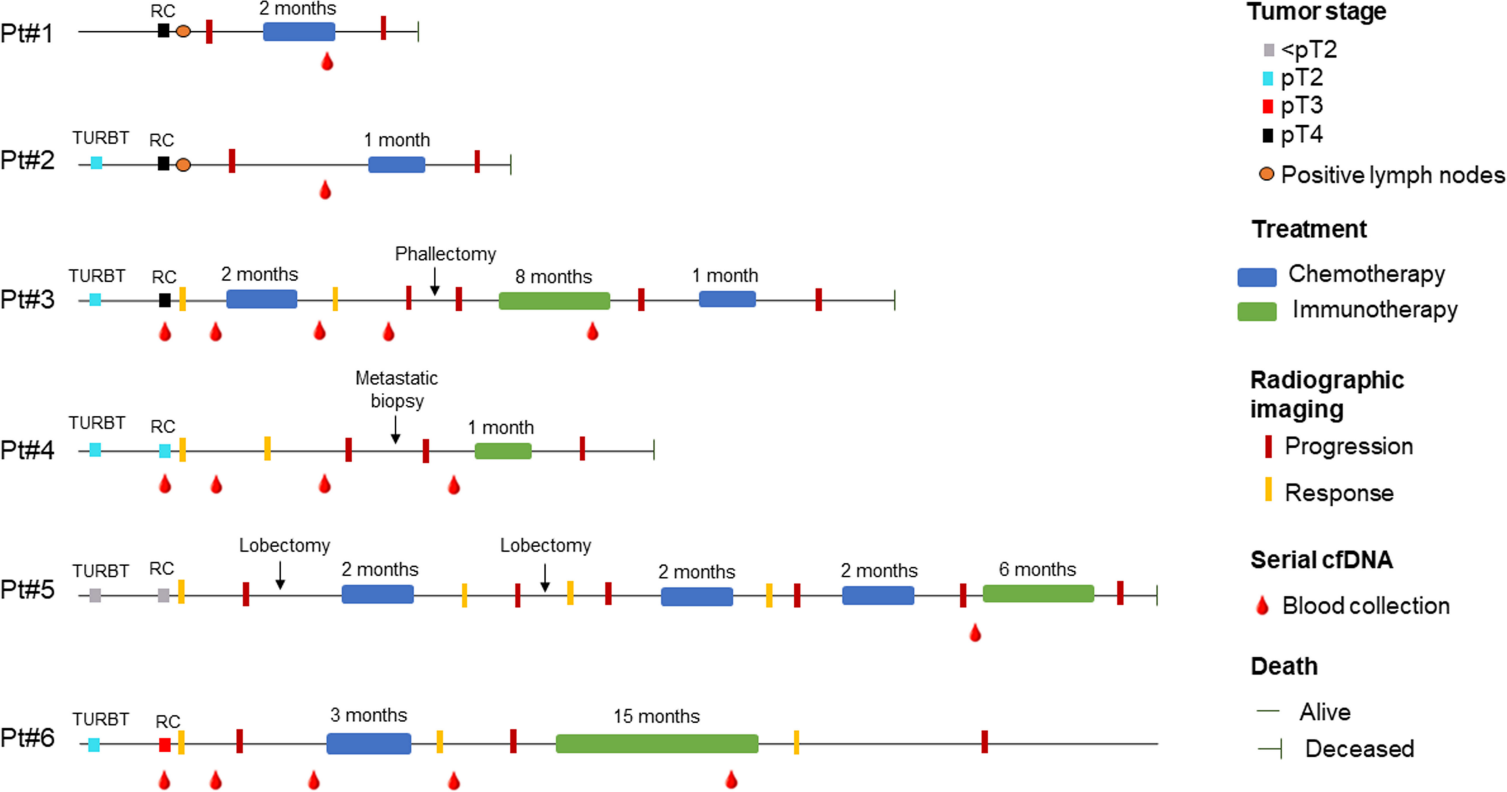
Clinicopathological variables and treatments received for the six patients included in the study at different follow-up time points. Clinical controls, treatments, and blood draws are indicated in chronological order; however, the temporal proportion between these events is not proportional along the chronological line. RC, radical cystectomy; TURBT, transurethral resection of bladder tumor.

The six patients enrolled in this study underwent radical cystectomy and lymphadenectomy, five due to MIBC and one (Pt#5) due to a T1 extensive tumor (TURBT). Two patients presented positive lymph nodes (LN+) at the time of cystectomy (Pt#1 and Pt#2). Clinical variables and treatments received during patient follow-up are summarized in **Figure 2**. IHC analysis determined that tumors from Pt#2, Pt#3, Pt#4, and Pt#6 presented basal differentiation, the tumor from Pt#5 had luminal differentiation, and the tumor from Pt#1 presented both luminal and basal subtypes. All six patients developed locoregional (Pt#1 and Pt#2) or distant metastases (Pt#3, Pt#4, Pt#5, and Pt#6). The median time to progression was seven months (range 1-22 months). One patient (Pt#3) received adjuvant chemotherapy, and all six patients received treatment upon tumor progression. Five of the six (83%) patients died due to BC; the median survival time was 16 months (range 5-93 months).

### 3.2 Mutations in primary tumor and metastatic samples

**Figure 3:**
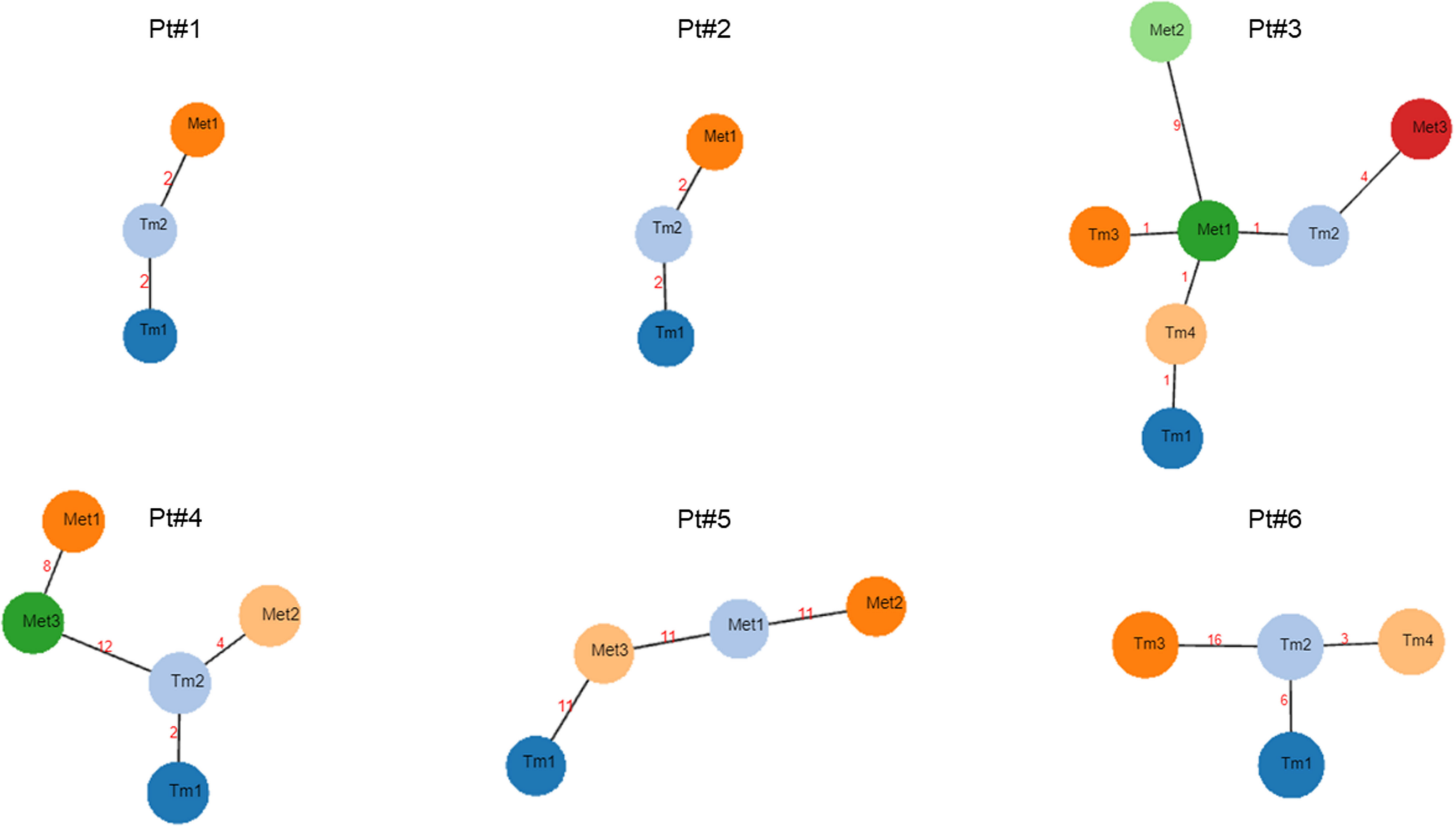
Phylogenetic trees showing tumor evolution for each metastatic bladder cancer patient. Each node represents a single primary tumor specimen or metastatic biopsy. The numbers between two nodes correspond to genetic changes between them. Met, metastatic biopsy; Pt, Patient; Tm, primary tumor biopsy.

The spatial molecular heterogeneity of BC was determined by examining several different morphologically tumor regions from the primary tumor and metastatic sites in each patient (**Figure 1**; **Supplementary Figure S1**). Each region harbored private as well as shared genetic alterations with the other different morphologically tumor regions (**Supplementary Table S5**), showing high BC intra- and inter-lesion heterogeneity but also intra- and inter-lesion shared genetic features. **Figure 3** depicts evolutionary trees displaying the evolution of somatic mutations in each patient. As shown, metastatic sites harbored additional mutations to the primary tumors, indicating an accumulation of somatic mutations over time (**Supplementary Table S5**).

Overall, a total of 6, 5, 9, 4, 21, and 42 mutations (mean of 15 mutations) were identified in the primary tumor samples from each of the six patients, respectively. Interestingly, primary tumors with basal differentiation (Pt#2, Pt#3, Pt#4, and Pt#6) harbored mutations in the key basal genes *RB1* and/or *TP53*. The tumor from Pt#5 had luminal differentiation and harbored *FGFR3* mutations. However, Pt#1, who presented a tumor with both molecular subtypes, did not harbor mutations in *FGFR3*, *RB1*, or *TP53*; instead, they harbored *KRAS* mutations (**Supplementary Table S5**).

Globally, a total of 7, 4, 25, 43, and 61 mutations (mean of 29 mutations) were identified in the metastatic sites of the first five patients, respectively. Of note, patients with distant metastases (Pt#3, Pt#4, and Pt#5) harbored a higher number of mutations than patients with locoregional metastases (Pt#1 and Pt#2) (mean of 43 vs. 6 mutations).

**Figure 4:**
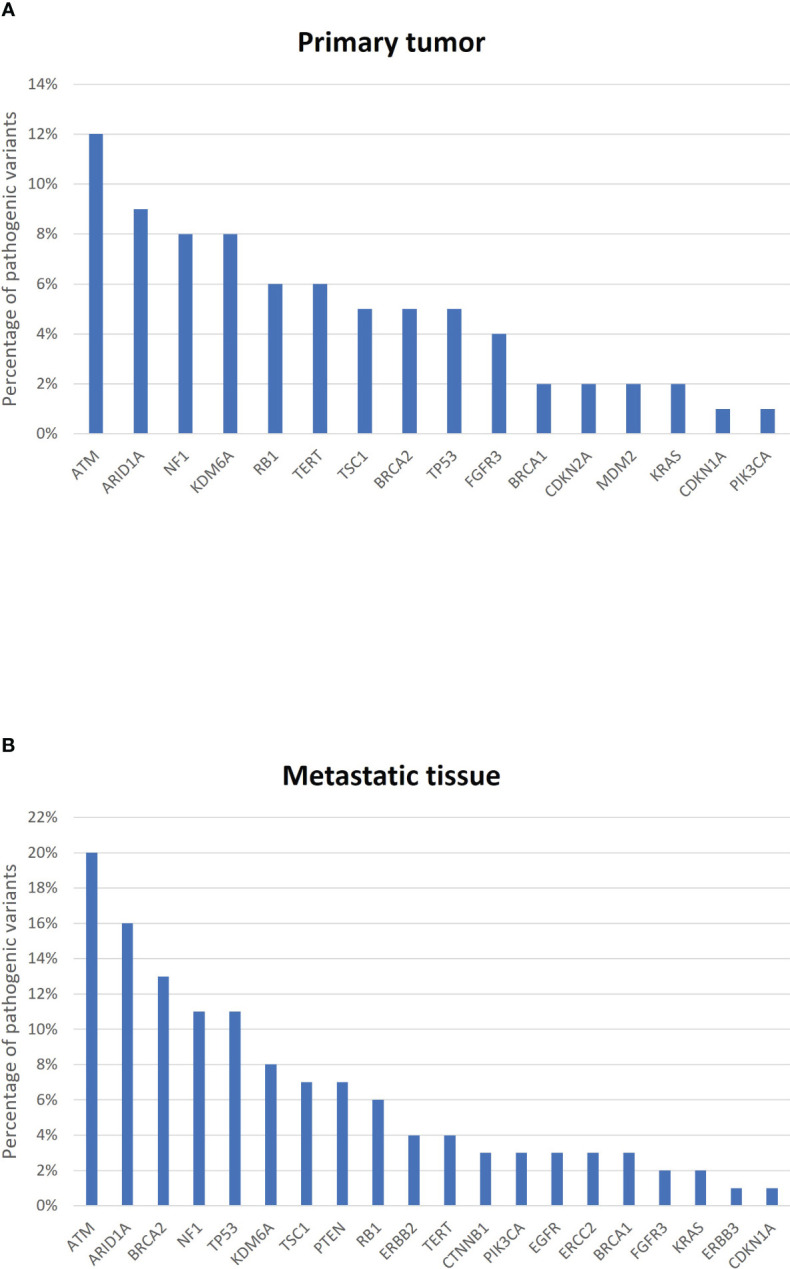
Most frequently mutated genes in **(A)** primary tumors and **(B)** metastatic sites from the six bladder cancer patients. The graph represents the percentage of pathogenic variants per gene.

The most frequently mutated genes in primary tumors were *ATM* (12%), *ARID1A* (9%), *NF1* (8%), and *KDM6A* (8%); in metastases, the genes were *ATM* (20%), *ARID1A* (16%), *BRCA2* (13%), *NF1* (11%), and *TP53* (11%) (**Figure 4**). Four mutations were identified in common between primary tumors and metastatic sites in the six BC patients (**Table 1**). Overall, the c.1-124C>T hotspot mutation in the *TERT* promoter was found in 60% (9/15) of primary tumor specimens and 55% (6/11) of metastatic sites. The *RB1* c.13delA mutation was found in 33% (5/15) of primary tumor regions and 36% (4/11) of metastatic regions. The *ATM* c.1236-2A>T mutation was also identified in 33% (5/15) of primary tumor samples but in only 9% (1/11) of metastatic samples. Finally, the *FGFR3* c.742C>T mutation was found in only 7% of primary tumor specimens and 27% of metastatic sites. Notably, these specific mutations were not found in all tumor regions from the same patient, confirming the spatial molecular heterogeneity of BC (**Table 1**).

**Table 1:** Distribution of the most frequent gene mutations according to different regions analyzed from the primary tumor and metastasis in each bladder cancer patient.

	Tumor biopsies	*TERT*	*ATM*	*RB1*	*FGFR3*
Pt#1	Tm_1.1				
	Tm_1.2				
	Met_1.1				
Pt#2	Tm_2.1				
	Tm_2.2				
	Met_2.1				
Pt#3	Tm_3.1				
	Tm_3.2				
	Tm_3.3				
	Tm_3.4				
	Met_3.1				
	Met_3.2				
	Met_3.3				
Pt#4	Tm_4.1				
	Tm_4.2				
	Met_4.1				
	Met_4.2				
	Met_4.3				
Pt#5	Tm_5.1				
	Met_5.1				
	Met_5.2				
	Met_5.3				
Pt#6	Tm_6.1				
	Tm_6.2				
	Tm_6.3				
	Tm_6.4				

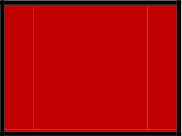
Presence of mutation in primary tumor.

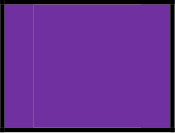
Presence of mutation in metastasis.

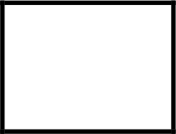
Absence of mutation.

Met, metastasis; Tm, primary bladder tumor.

Specific mutations: TERT c.1-124C>T; ATM c.1236-2A>T; RB1 c.13delA; FGFR3 c.742C>T.

### 3.3 Mutation analysis in cfDNA and correlation with tumor tissue

ddPCR was used to determine the four mutations (*TERT* c.1-124C>T, *RB1* c.13delA, *ATM* c.1236-2A>T, and *FGFR3* c.742C>T) shared between the primary tumors and their respective metastases in plasma cfDNA samples. **Table 2** depicts mutations identified in tumor tissue (primary and metastasis) and cfDNA at different follow-up time points in the six BC patients. As shown, mutations in metastatic tissue and cfDNA samples during patient follow-up had a higher concordance than mutations in primary tumors and cfDNA samples at the time of cystectomy (89% vs. 29%). Furthermore, mutation evolution is evidenced by their dynamic changes during patient follow-up. For instance, Pt#3 was treated with three cycles of adjuvant gemcitabine-cisplatin chemotherapy after RC. Both *TERT* and *RB1* mutations were present in their cfDNA at the time of RC, but the *RB1* mutation was not detected after chemotherapy (4 and 12 months after RC). In Pt#6, the *RB1* mutation was identified prior to four cycles of salvage gemcitabine-cisplatin chemotherapy (four months after RC), but was absent after treatment (12 months after RC) (**Figure 2**; **Table 2**).

**Table 2:** Detection of specific gene mutations in tumor biopsies and cell-free DNA (cfDNA) samples during patient follow-up.

		Tumor biopsies		cfDNA samples					
		Tm	Met	RC	1m	4m	12m	24m	Xm
Pt#1	*TERT*			–	–				
	*ATM*			–	–				
Pt#2	*TERT*			–	–				
	*ATM*			–	–				
Pt#3	*TERT*								
	*RB1*								
	*TERT*								
Pt#4	*ATM*								
	*RB1*								
Pt#5	*RB1*			–	–	–	–	–	
	*FGFR3*			–	–	–	–	–	
Pt#6	*ATM*		–						
	*RB1*		–						

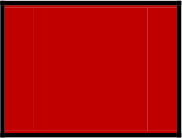
Presence of mutation.

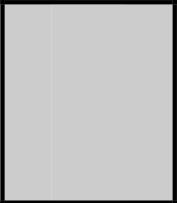
Absence of mutation.

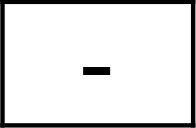
Sample not available.

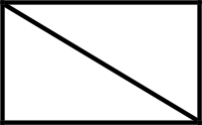
Exitus.

Met, metastasis; RC, radical cystectomy; Tm, primary bladder tumor; 1m, 1 month after RC; 4m, 4 months after RC; 12m, 12 months after RC; 24m, 24 months after RC; Xm, 84 months (7 years) after RC.

Specific mutations: TERT c.1-124C>T; ATM c.1236-2A>T; RB1 c.13delA; FGFR3 c.742C>T.

## 4 Discussion

Bladder cancer is one of the tumors with the highest genetic heterogeneity (4), presenting a challenge to identify a set of recurrent cfDNA biomarkers for use in the clinical setting to monitor tumor evolution in response to treatment (12).

Here, we have addressed the challenge of BC heterogeneity by analyzing genetic features of several different morphologically tumor regions from primary bladder tumor and locoregional/distant metastases from six BC patients. Furthermore, we have identified genetic alterations shared between primary bladder tumors and their metastases in different BC patients and we have been able to determine them in plasma cfDNA from these patients during their follow-up. The assessment of these BC genetic alterations providing aggressive behavior in plasma cfDNA would allow real-time monitoring of tumor evolution in a real clinical scenario.

Since some of the MIBC molecular subtypes have been associated with bladder tumor aggressivity, we first classified bladder tumors included in this study according to basal or luminal differentiation. As expected, the four MIBC tumors from our cohort that presented basal differentiation harbored mutations in the *TP53* and/or *RB1* genes. This subtype has been associated with aggressive tumors and a poor prognosis (13, 14) and indeed, these patients did have a poor prognosis, presenting a two-year mortality rate of 75%. The tumor from Pt#5 with luminal differentiation harbored an *FGFR3* mutation, as expected for this molecular subtype. This is the only non-muscle-invasive BC patient in our series and the one with the longest progression-free survival (seven years). In fact, BC patients with luminal tumors have been found to have the best prognosis compared with all other molecular subtypes (13, 14). Finally, BC tumors with both basal and luminal subtypes have been suggested to present aggressive behavior since they are considered to have a similar prognosis to basal subtype (13, 14) and actually Pt#1 showed a short cancer-specific survival. This tumor did not harbor mutations in the *TP53*, *RB1*, or *FGFR3* genes. However, we found genomic alterations in *KRAS*, promoting the activation of the RAS pathway in this tumor, which has been suggested to be associated with luminal differentiation (14, 15).

Germane to the mutations present in the distinct tumor regions of primary bladder tumors as well as locoregional/distant metastases, we were able to confirm previously reported ITH of bladder tumors, as well as lesion-to-lesion genetic heterogeneity (12, 16, 17). In fact, phylogenetic trees showing branched tumor evolution in each patient demonstrated the existence of more aggressive clones in primary tumors that would originate the metastasis. Additionally, we found that distant metastases were genetically more altered than locoregional ones, and both metastatic regions were genetically more altered than primary tumors, most probably due to the emergence of resistance mutations during disease progression (18). Interestingly, although both high genetic intra- and inter-lesion heterogeneity were identified, we were able to detect several mutations in common between primary tumors and metastatic lesions. All six patients harbored at least one of these four mutations in primary tumors and metastatic lesions: *TERT* c.1-124C>T, *ATM* c.1236-2A>T, *RB1* c.13delA, and *FGFR3* c.742C>T. These inter-lesion-shared mutations were analyzed in cfDNA samples during patient follow-up for real-time monitoring of disease relapse. Remarkably, a higher DNA mutation concordance was found between metastatic lesions and cfDNA samples during follow-up than between the primary tumor and cfDNA at the time of cystectomy. Multiple factors can account for this DNA mutation discordance between tumor and cfDNA at surgery, such as preanalytical methodological factors (amount of plasma analyzed, assay sensitivity, etc.) (10) or low cfDNA levels that could prevent variant detection (false negative). In fact, other authors previously reported better concordance between cfDNA and tissue samples when high cfDNA levels were present (19).

Substantial evidence suggests that the detection of circulating tumor DNA (ctDNA; the variable fraction of plasma cfDNA derived from tumor) in the bloodstream following potential curative treatment is associated with a high risk of tumor relapse (7, 11, 20–22). However, the identification of specific mutations in cfDNA can also be an advantage for applying targeted therapy. For instance, those patients with advanced or metastatic BC harboring *FGFR3* mutations in their primary tumor are already being treated with the Food and Drug Administration (FDA)-approved drug Erdafitinib^®^ (NCT05316155), a fibroblast growth factor receptor (FGFR) kinase inhibitor (23). Interestingly, an *FGFR3* missense mutation (c.742C>T; p.Arg248Cys), a gain-of-function mutation that promotes cell proliferation and migration (24, 25), was found in Pt#5’s cfDNA samples, suggesting that liquid biopsy could be a useful tool to identify potential therapeutic targets during patient follow-up. In fact, this specific *FGFR3* mutation was also identified in cfDNA samples from a series of BC patients who were candidates for Erdafitinib (12), emphasizing that the detection of the *FGFR3* mutation in plasma cfDNA could be helpful to guide targeted treatment in a non-invasive way. *FGFR3* alterations occur mainly in non-muscle-invasive BC (25), and it was mutated in the only patient of our series (Pt#5) with non-invasive disease at the time of RC. However, this patient did not present an *RB1* mutation in their primary bladder tumor; however, they did in their metastatic tissue. This is most likely due to *RB1* mutations being commonly found in higher BC stages and associated with poor patient outcomes (26).

As regards somatic mutations in DNA repair genes, such as *ATM* and *RB1*, these have been associated with better responses to cisplatin-based chemotherapy in MIBC (27–29). Interestingly, three patients from our cohort (Pt#3, Pt#4, and Pt#5) harbored an *RB1* mutation both in primary tumors/metastatic sites and plasma cfDNA. This mutation was undetectable in cfDNA from Pt#3 and Pt#5 after treatment with gemcitabine-cisplatin chemotherapy. The remaining patient (Pt#4) did not receive cisplatin-based chemotherapy, and the *RB1* mutation was present in their cfDNA during the entire follow-up. *RB1* is a tumor suppressor gene that has been associated with DNA repair as well as cell proliferation and division (27). Several studies have demonstrated that mutations in *RB1* are frequently found in bladder cancer (26, 27), although, as far as we know, the specific mutation (c.13delA; loss-of-function mutation) found in our cohort had not been described before. On the other hand, *ATM* is a core component of the DNA repair system, and its signaling pathway is known to play a role in the development of breast cancer and other tumors (30). Several mutations in *ATM* have been previously described in BC (26, 31), however, interestingly, the specific ATM mutation found in our cohort (c.1236-2A>T) was first described in our previous study analyzing bladder tumors (32). Finally, the *TERT* promoter mutation (c.1-124C>T, gain-of-function mutation) has been widely reported in several solid tumors, including bladder cancer (33–36), where it plays an important role in tumorigenesis. In a previous study (22), which included both progressive and non-progressive BC patients, we showed that none of the non-progressive patients harboring *ATM* and *TERT* mutations in their primary tumors, present these mutations in their cfDNA during their follow-up after RC. Furthermore, in another previous study, we described that the presence of the mutation *TERT* c.1-124C>T could be considered a biomarker of aggressivity in MIBC (32). This data suggests that the presence of mutation in *ATM* and *TERT* in plasma cfDNA after treatment is related to tumor dissemination and confers a poor prognosis to patients.

To the best of our knowledge, this is the first work to evaluate in plasma cfDNA a set of genetic alterations associated with aggressive behavior in bladder cancer, initially identified in primary and metastatic sites from BC patients. Nonetheless, we must acknowledge some study limitations. First, some tumors might not have shed ctDNA into the plasma at the time of cystectomy, even if the tumor had a targetable mutation, with the consequent risk of a false-negative result. In addition, this study includes only six patients. However, it must be considered the difficulty of obtaining metastatic biopsies from these patients. In fact, our cohort of metastatic patients is one of the largest in the literature considering the type of samples analyzed from each patient (different morphologically tumor regions from primary and metastatic sites). Nonetheless, more patients need be investigated to determine the final role of these four genes as biomarkers for monitoring tumor evolution and assessment of treatment response in advanced, metastatic BC patients.

## 5 Conclusion

A higher number of mutations was identified in distant metastases compared with locoregional metastases. Moreover, mutations in metastatic tissue had a high concordance with mutations in cfDNA samples. Despite BC genetic intra- and inter-lesion heterogeneity, specific mutations in *TERT*, *ATM*, *RB1*, and *FGFR3* were found to be shared between primary and metastatic lesions in different BC patients. These shared mutations have been identified in cfDNA samples during patient follow-up, allowing real-time monitoring of these patients. Consequently, the analysis of BC mutations associated with poor prognosis in plasma cfDNA could be a valuable tool to monitor tumor evolution in response to treatments, thus improving the clinical management of BC patients.

## Data availability statement

The datasets presented in this study can be found in online repositories. The names of the repository/repositories and accession number(s) can be found in the article/**Supplementary Material**.

## Ethics statement

The studies involving humans were approved by Clinical Research Ethics Committee of the Hospital Clinic of Barcelona (HCB/2018/0026). The studies were conducted in accordance with the local legislation and institutional requirements. The participants provided their written informed consent to participate in this study.

## Author contributions

RC: Investigation, Methodology, Writing – original draft. MI-T: Resources, Writing – review & editing. JO: Resources, Writing – review & editing. FR: Writing – review & editing. LR-C: Methodology, Writing – review & editing. SH: Writing – review & editing. BM: Data curation, Writing – review & editing. AA: Writing – review & editing. LI: Writing – review & editing. LM: Funding acquisition, Investigation, Supervision, Writing – review & editing.
