# Supplementary material for: Assessment of aggressive bladder cancer mutations in plasma cell-free DNA
Source: Front Oncol. 2023 Nov 30;13:1270962. doi: 10.3389/fonc.2023.1270962 (PMC10720633; doi:10.3389/fonc.2023.1270962)
Supplement: Supplementary file 1 [file DataSheet_1.pdf]

Supplementary Material

1 Supplementary Methods

- Determination of limit of detection (LOD)

Wild-type (wt) and mutant alleles for each assay were obtained by cloning each gene region containing every allele. Cloning experiments were performed according to our previous study (1).

An optimal annealing temperature was identified for each of the four genes; 10,000 copies of the wt allele were mixed with 10,000 copies of the mutant allele, and droplet digital PCR (ddPCR) was performed at eight different annealing temperatures (range: 53ºC to 63ºC) to identify the optimal temperature at which the wt and mutant droplets are sufficiently separated. The optimal annealing temperature for each assay was: 60ºC for RB1 c.13delA, 59ºC for FGFR3 c.742C>T, 60ºC for TERT c.1-124C>T, 56.5ºC for ATM. Subsequently, we detected the LOD for each assay performing ddPCR with 10,000 copies of wt allele and different copies of mutant allele as indicated in the following table:

Wild-types copies	Mutant copies
10,000	5,000
10,000	2,500
10,000	1,000
10,000	500
10,000	250
10,000	150
10,000	100
10,000	50
10,000	10
10,000	5
10,000	1
10,000	0

The LOD of the mutant allele was 0.05% for RB1, FGFR3, and ATM, and 0.08% for TERT.

- ddPCR

Reactions were performed with 11 µl SuperMix no dUTP (Bio-Rad), 0.5 µl forward + reverse primers (40 µM), 1.1 µl HEX probe (5 µM), 1.1 ul FAM probe (5 µM) and 8.3 µl cfDNA sample. PCR was performed at 95ºC for 10 min, followed by 40 cycles (except for TERT c.1-124C>T, which was 50 cycles) at 94ºC for 30 seconds, the specific assay annealing temperature for 1 min, followed by 98ºC for 10 min, and a final hold at 4ºC. Each reaction was performed with a negative control (10,000 copies wt allele + 0 copy mutant allele) and positive control (10,000 copies wt allele + 10 copies mutant allele).

ctDNA was defined as detectable if the mutant allele fraction (MAF) was above the LOD of each mutant amplicon.

REFERENCES

1. Carrasco R, Ingelmo-Torres M, Gómez A, Trullas R, Roldán FL, Ajami T, Moreno D, Rodríguez-Carunchio L, Alcaraz A, Izquierdo L, et al. Cell-Free DNA as a Prognostic Biomarker for Monitoring Muscle-Invasive Bladder Cancer. Int J Mol Sci (2022) 23:11732. doi: 10.3390/ijms231911732

2. Supplementary Figures

Supplementary Figure S1. Biopsies were obtained from primary tumors and locoregional/distant metastases from each BC patient. Each image corresponds to core regions harvested from RC/TURBT and metastasectomy specimens at different scales. Different cell types and morphologies in each tumor sample are shown with hematoxylin-eosin staining. Two primary tumor images (one from Pt#3 and another from Pt#4) were not available.

(See PDF file).

Abbreviations: Met; metastatic specimen, Pt; patient, RC; radical cystectomy, Tm; primary tumor specimen, TURBT; transurethral resection of bladder tumor.

3. Supplementary Tables

Supplementary Table S1. Clinicopathological features and treatment received for the six metastatic BC patients.

	Pt#1	Pt#2	Pt#3	Pt#4	Pt#5	Pt#6
Gender	M	M	M	M	F	F
Age at diagnosis	67	61	58	78	74	75
**Radical cystectomy**						
pT stage	pT4N3	pT4N3	pT4N0	pT2N0	pTaN0	pT3N0
Grade	G3	G3	G3	G3	G3	G3
N status	N1	N1	N0	N0	N0	N0
Focality	Multi	Multi	Multi	Uni	Multi	Uni
Adjuvant Chemotherapy	-	-	Gemcitabine-cisplatin	-	-	-
**Progression**						
Time to progression (mo)	1	3	16	10	22	4
Metastatic sites	Liver, bone	Liver, lung	Penis, lung, liver	Ileal Intestine/Cecum, Liver	Lung, bone	Pelvis
Salvage Chemotherapy	Carboplatin	Carboplatin	Carboplatin + immunothera py (Atezolizuma b)	Immunotherapy (anti-PD1)	Gemcitabine-cisplatin + immunotherapy (Pembrolizuma b)	Gemcitabine-cisplatin + immunotherapy (Atezolizumab)
Time to death (mo)	5	10	30	16	93	-
CSM	Yes	Unknown	Yes	Yes	Yes	No
Follow-up (mo)	5	10	30	16	93	43
**Type of tissue analyzed**						
Primary tumor	Cystectomy specimen	Cystectomy specimen	Cystectomy specimen	Cystectomy specimen	TURBT specimen	Cystectomy specimen
Metastatic site	Lymph node	Lymph node	Penis	Ileal Intestine	Lung	-

Abbreviations: CSM; cancer-specific mortality, Mo; months, Pt; patient, RC; radical cystectomy.

Supplementary Table S2. Genes from the Oncomine Bladder Panel (Thermo Fisher).

BLADDER CORE GENES PANEL	ADDITIONAL GENES
AKT1	*BRCA1*
ARID1A	*BRCA2*
ATM	*EGFR**
*BRAF**	*MYC*
*CCND1**	*NF1*
*CCNE1**	*TERT*
CDKN1A	
CDKN2A	
*CTNNB1**	
E2F3	
*ERBB2**	
*ERBB3**	
ERCC2	
*FGFR2**	
*FGFR3**	
*HRAS**	
KDM6A	
*KRAS**	
MDM2	
PIK3CA	
PPARG	
PTEN	
RB1	
TP53	
TSC1	

*Hotspot genes

Table S3. Plasma mutations analyzed in each patient.

	TERT c.1-124C>T	*ATM* c.1236-2A>T	*RB1* c.13delA	*FGFR3* c.742C>T
Pt#1	X	X		
Pt#2	X	X		
Pt#3	X		X	
Pt#4	X	X	X	
Pt#5			X	X
Pt#6		X	X	

Abbreviations: Pt; patient.

Supplementary Table S4. Primers and probes used for droplet digital PCR.

Primers and Probes	Company	Custom-designed
RB1<br><br>(c.13delA)	IDT	Forward primer seq:<br><br>CGCTCCTCCACAGCTCGCT<br><br>Reverse primer seq:<br><br>TCCTGCTCTGGGTCCTCC<br><br>Mutant probe:<br><br>ATGCCGCCCAAACCCCC (FAM)<br><br>Wild-type probe:<br><br>ATGCCGCCCAAAACCCCC (HEX)
FGFR3<br><br>(c.742C>T)	IDT	Forward primer seq:<br><br>AGTGGCGGTGGTGGTGAG<br><br>Reverse primer seq:<br><br>AGCACCGCCGTCTGGTTG<br><br>Mutant probe:<br><br>CACAGAGTGCTCCCCGCA (FAM)<br><br>Wild-type probe:<br><br>CACAGAGCGCTCCCCGCA (HEX)
TERT<br><br>(c.1-124C>T)	IDT	Forward primer seq:<br><br>CTCCTCCGCGCGGCACCCG<br><br>Reverse primer seq:<br><br>AAACTCGCGCCGCGAGGA<br><br>Mutant probe:<br><br>CAGCCCCTTCCGGGCCCT (HEX)<br><br>Wild-type probe:<br><br>CGACCCCCTCCGGGCCCT (FAM)

ATM<br><br>(c.1236-2A>T)	IDT	Forward primer seq:<br><br>TCCTTTTAGTTTGTTAATGTGATGGA<br><br>Reverse primer seq:<br><br>TCATCAGTAATGGAGACAGCTCA<br><br>Mutant probe:<br><br>TGGCTACAGATTGCAACCCA (HEX)<br><br>Wild-type probe:<br><br>AGGCTACAGATTGCAACCCA (FAM)

Supplementary Table S5. Pathogenic variants found in each tumor biopsy from each BC patient analyzed by NGS.

(See Excel file).

Abbreviations: CNV; copy number variation, INDEL; insertion/deletion polymorphism, Met; metastatic specimen, Pt; patient, SNV; single nucleotide variant, Tm; primary tumor specimen.
