# Supplementary figures and images for: Assessment of aggressive bladder cancer mutations in plasma cell-free DNA
Source: Front Oncol. 2023 Nov 30;13:1270962. doi: 10.3389/fonc.2023.1270962 (PMC10720633; doi:10.3389/fonc.2023.1270962)

**P#1**

**Tm\_1.1**

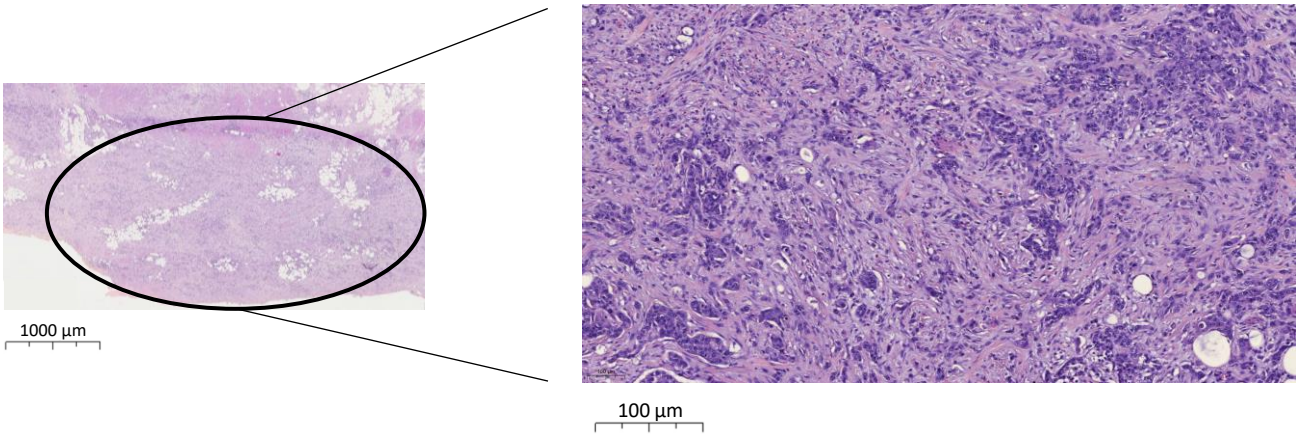

**Tm\_1.2**

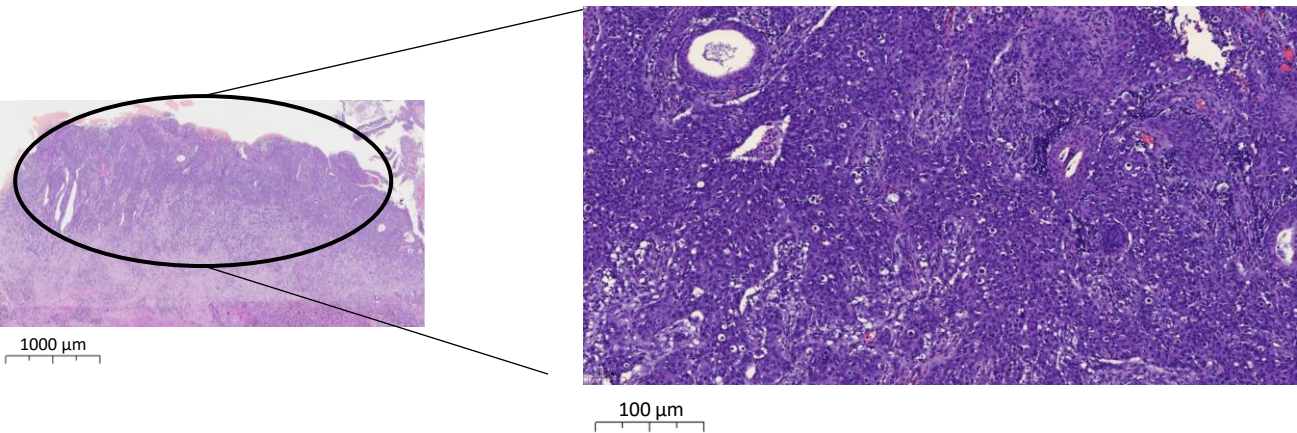

**Met\_1.1**

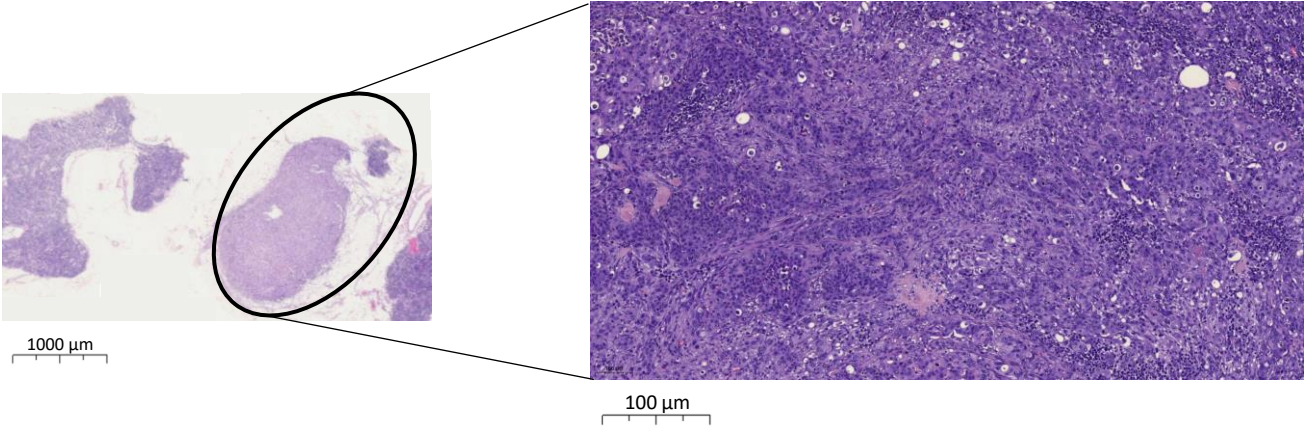

**Pt#2**

**Tm\_2.1**

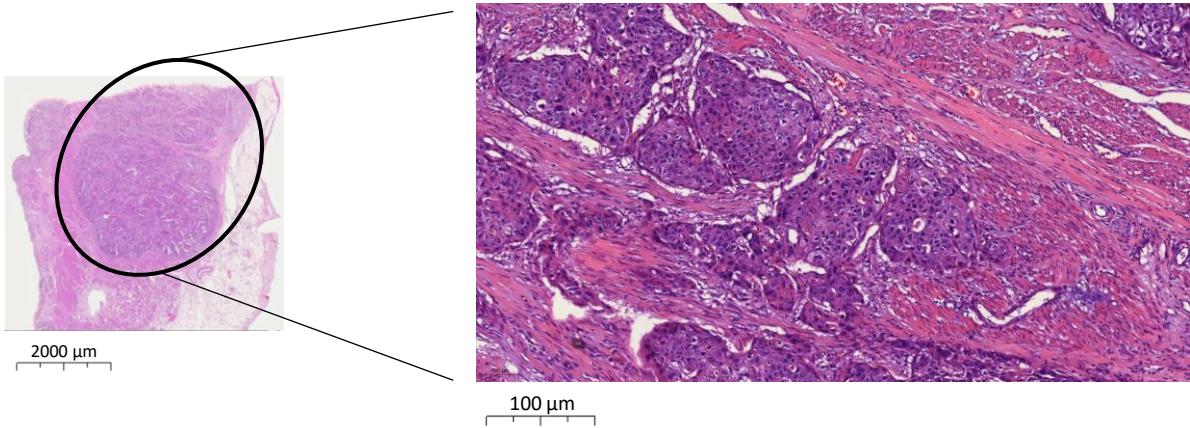

**Tm\_2.2**

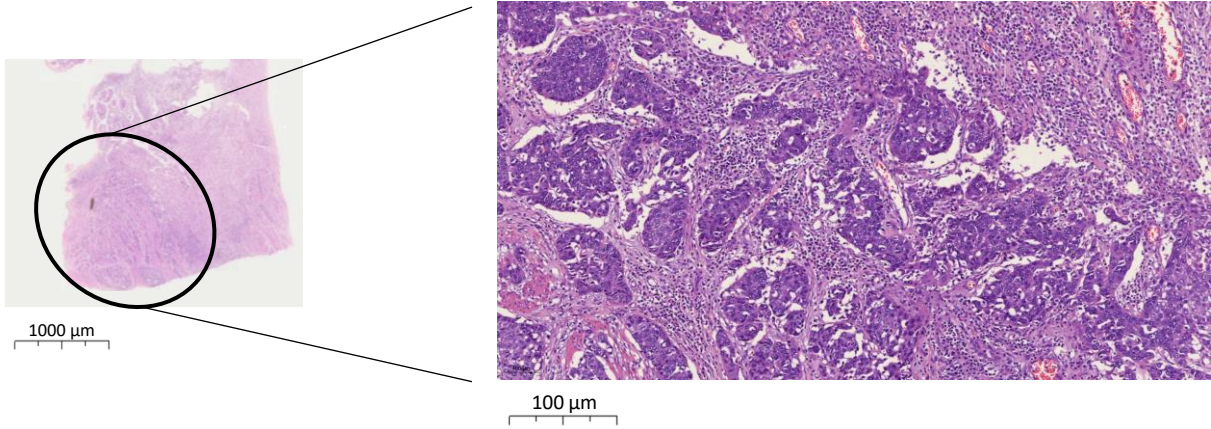

**Met\_2.1**

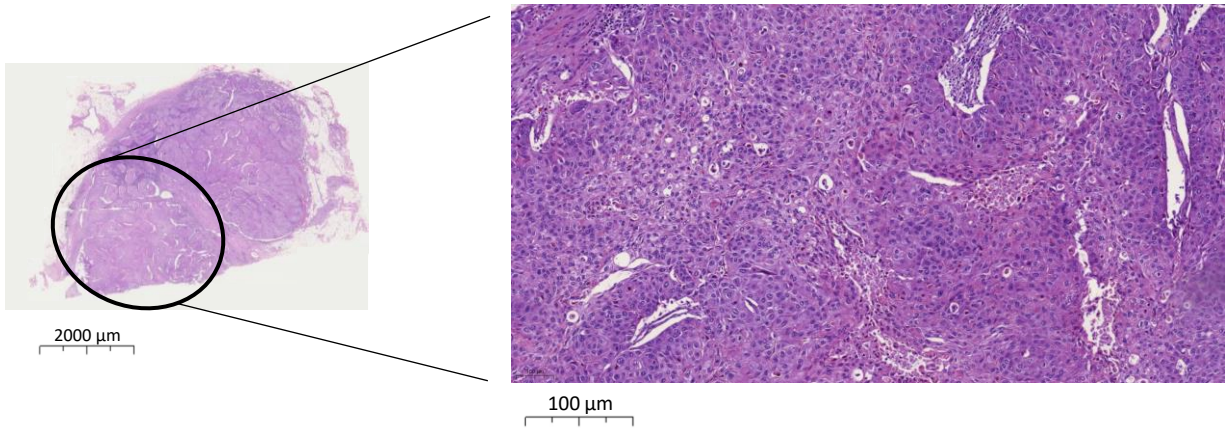

**Pt#3**

**Tm\_3.1**

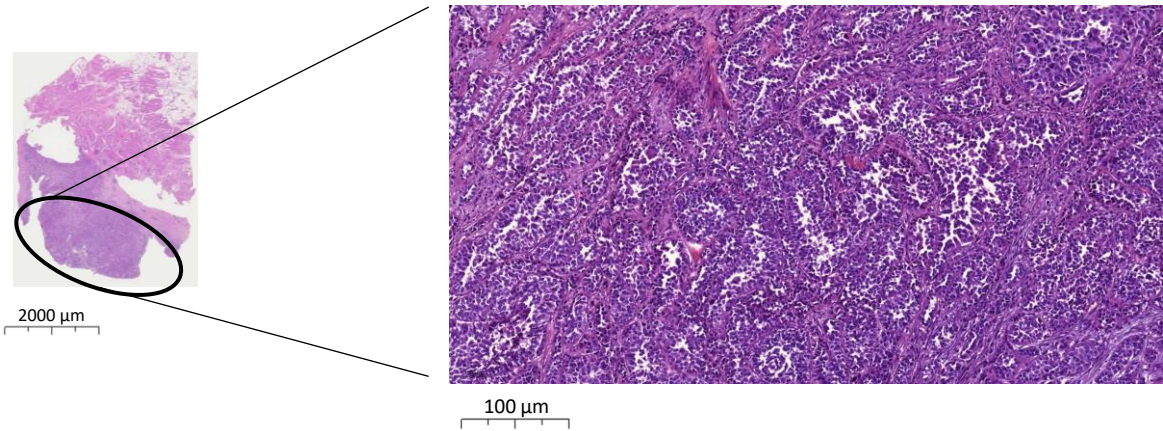

**Tm\_3.2**

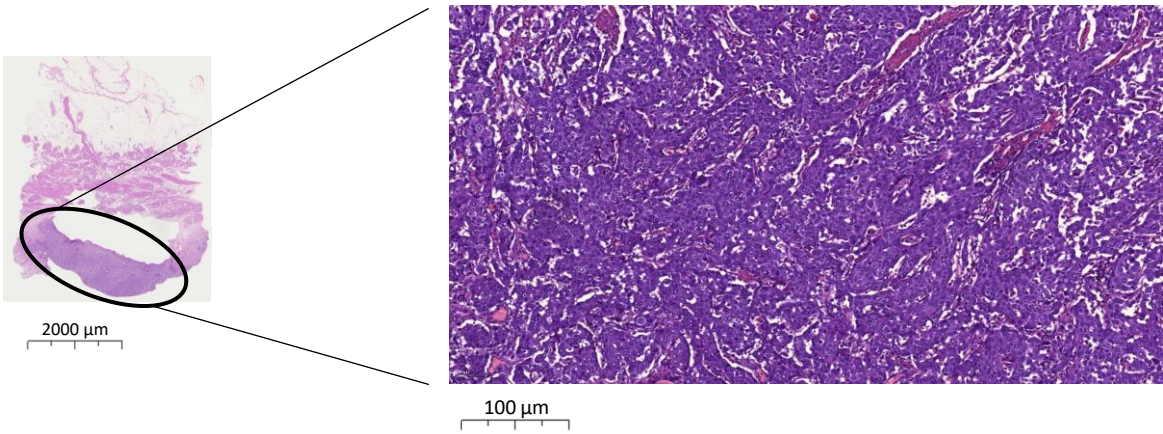

**Tm\_3.3**

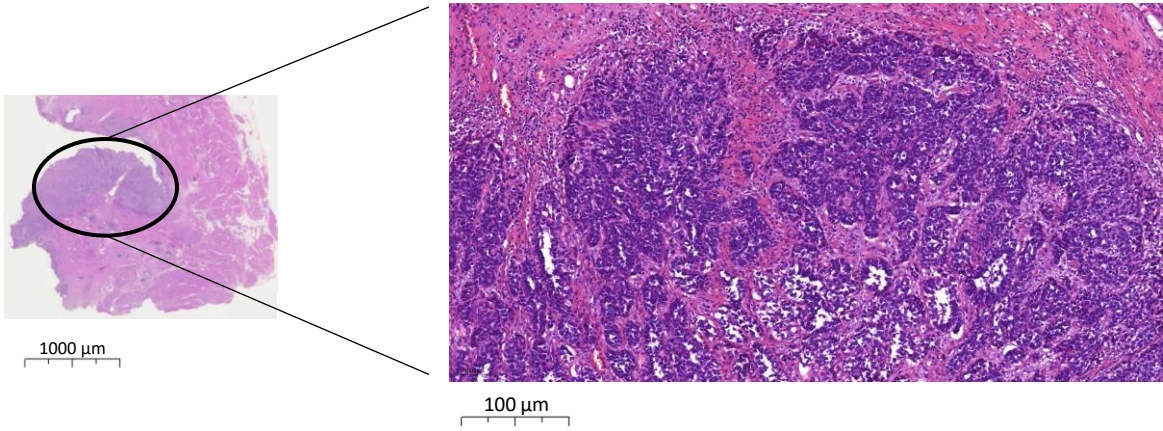

**Pt#3**

**Met\_3.1**

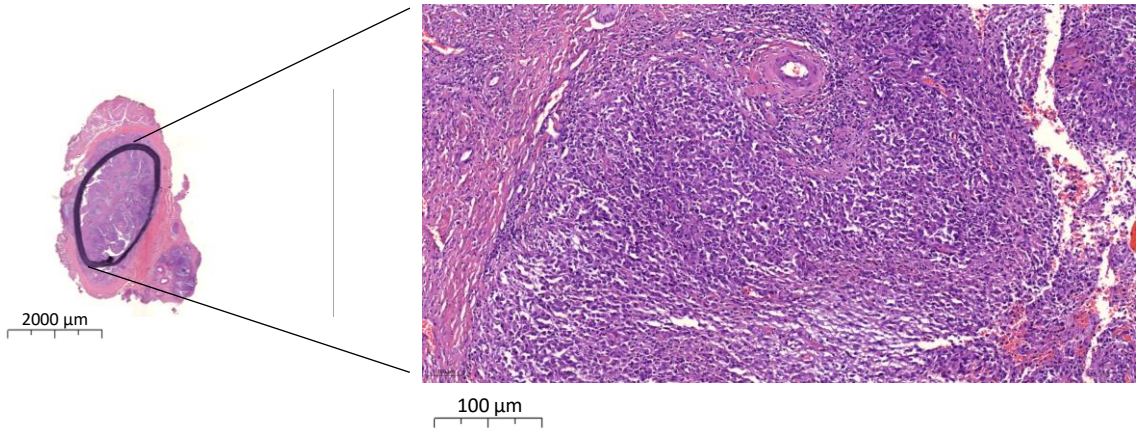

**Met\_3.2**

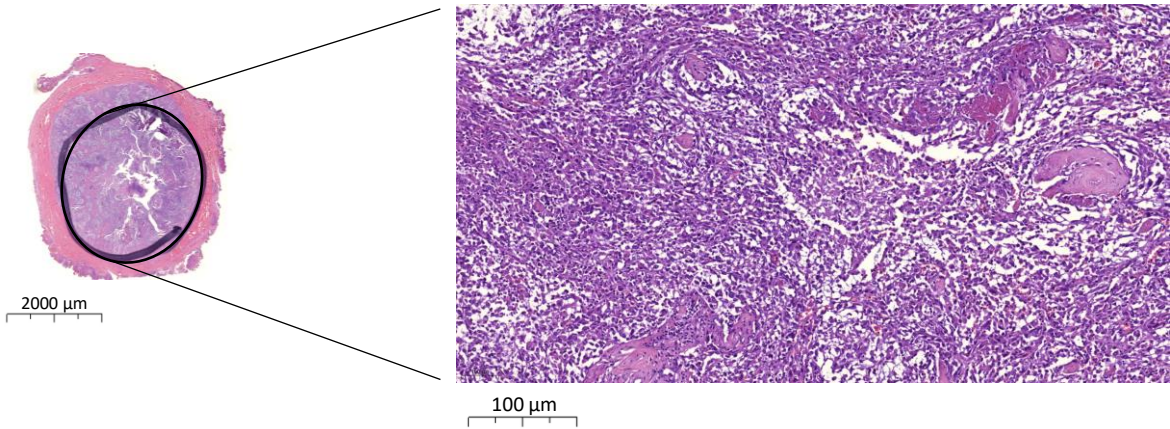

**Met\_3.3**

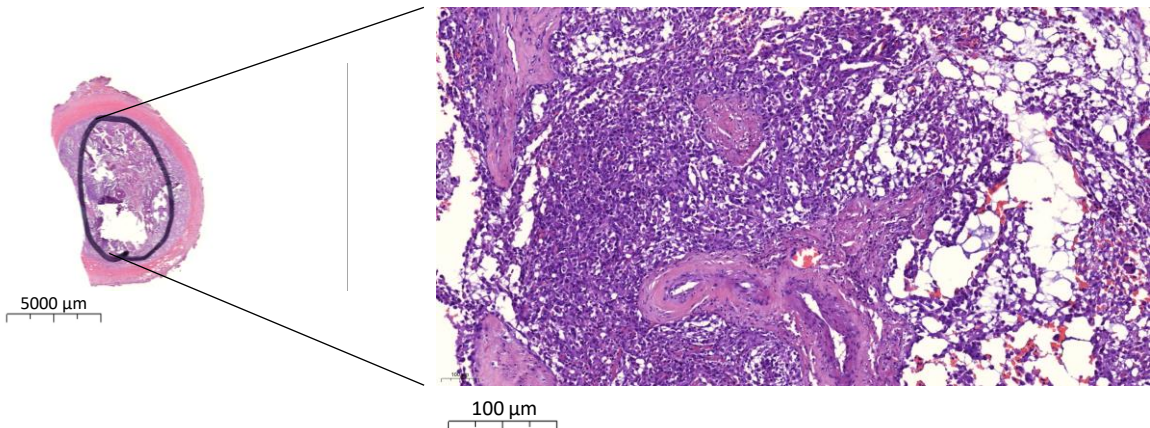

**Pt#4**

**Tm\_4.1**

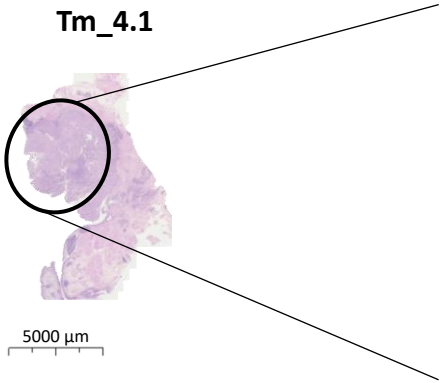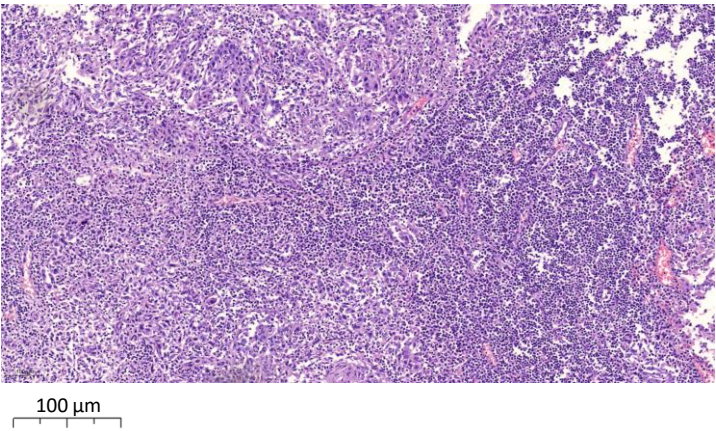

**Met\_4.1**

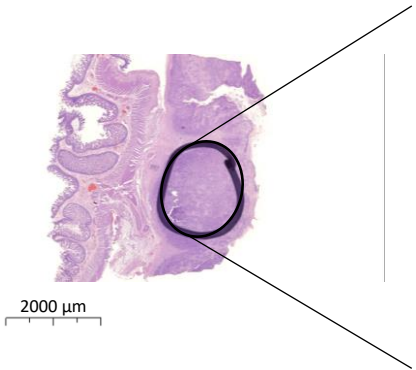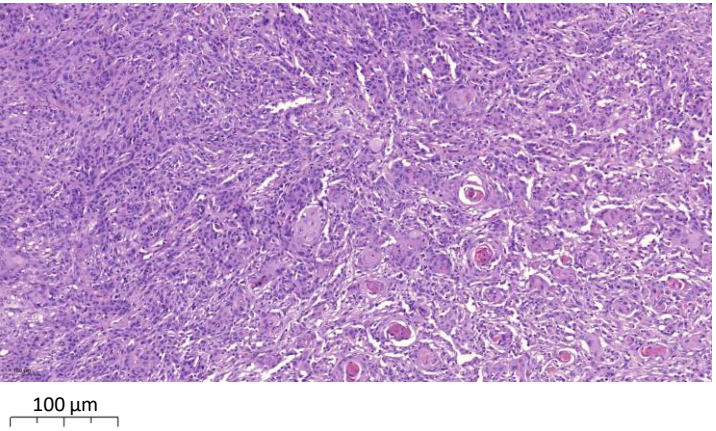

**Met\_4.2**

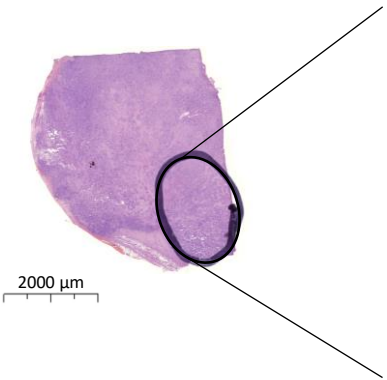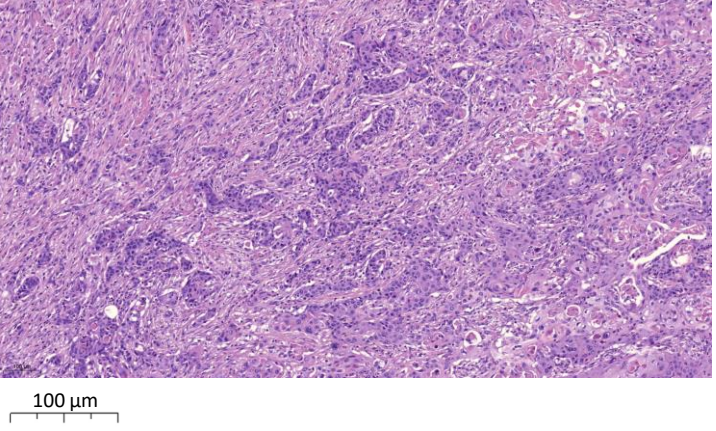

**Met\_4.3**

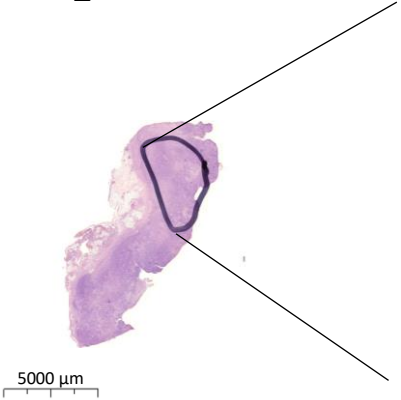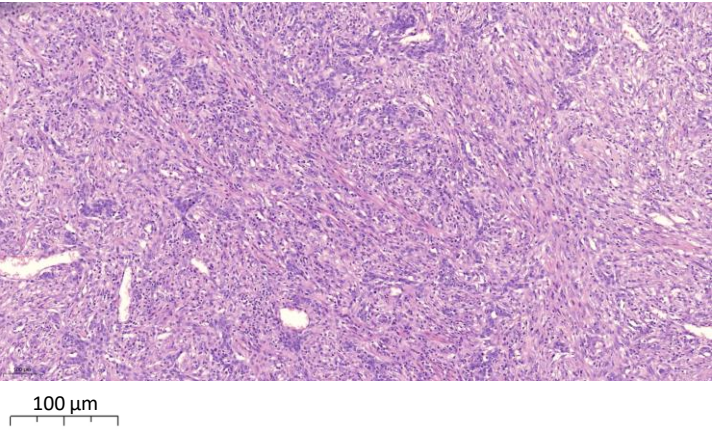

**Pt#5**

**Tm\_5.1**

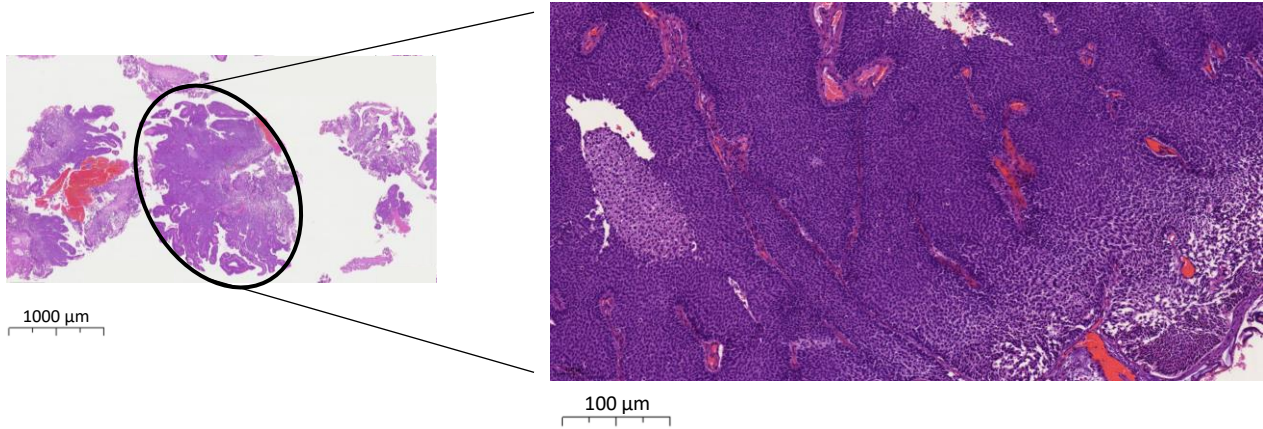

**Met\_5.1**

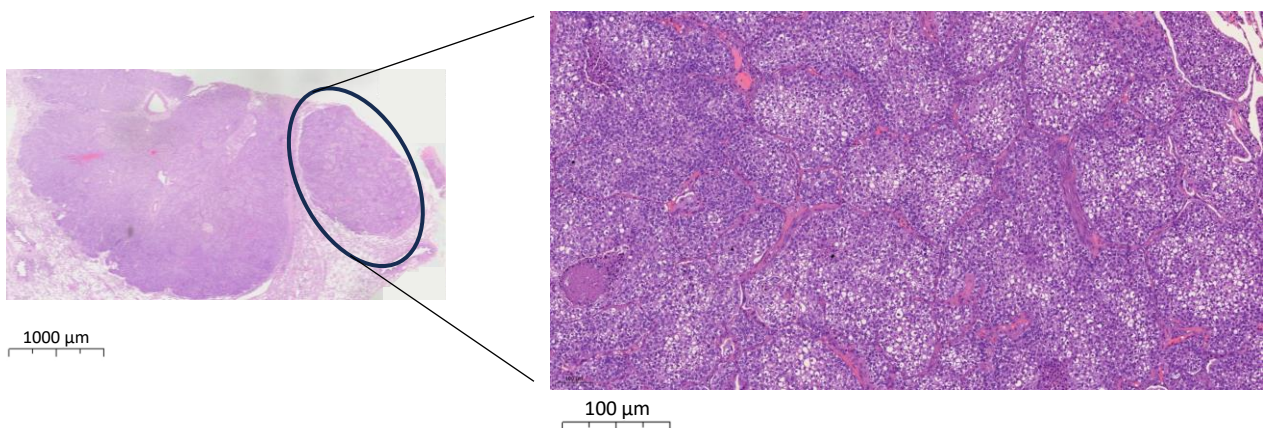

**Met\_5.2**

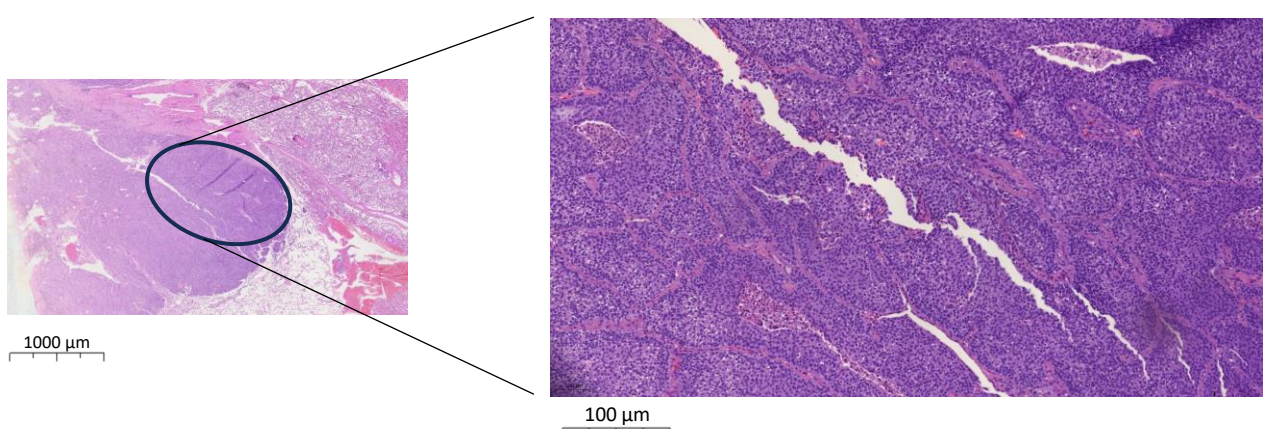

**Met\_5.3**

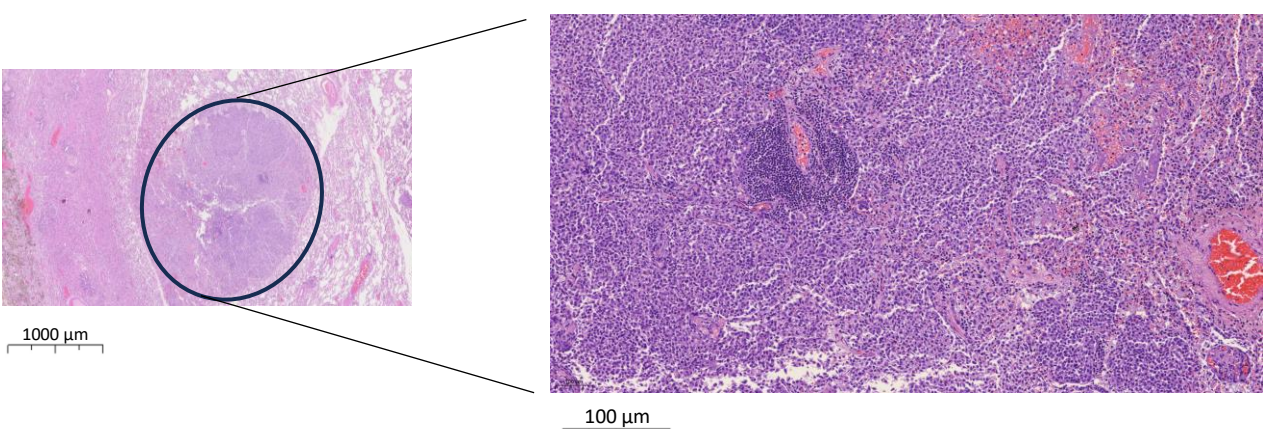

**Pt#6**

**Tm\_6.1**

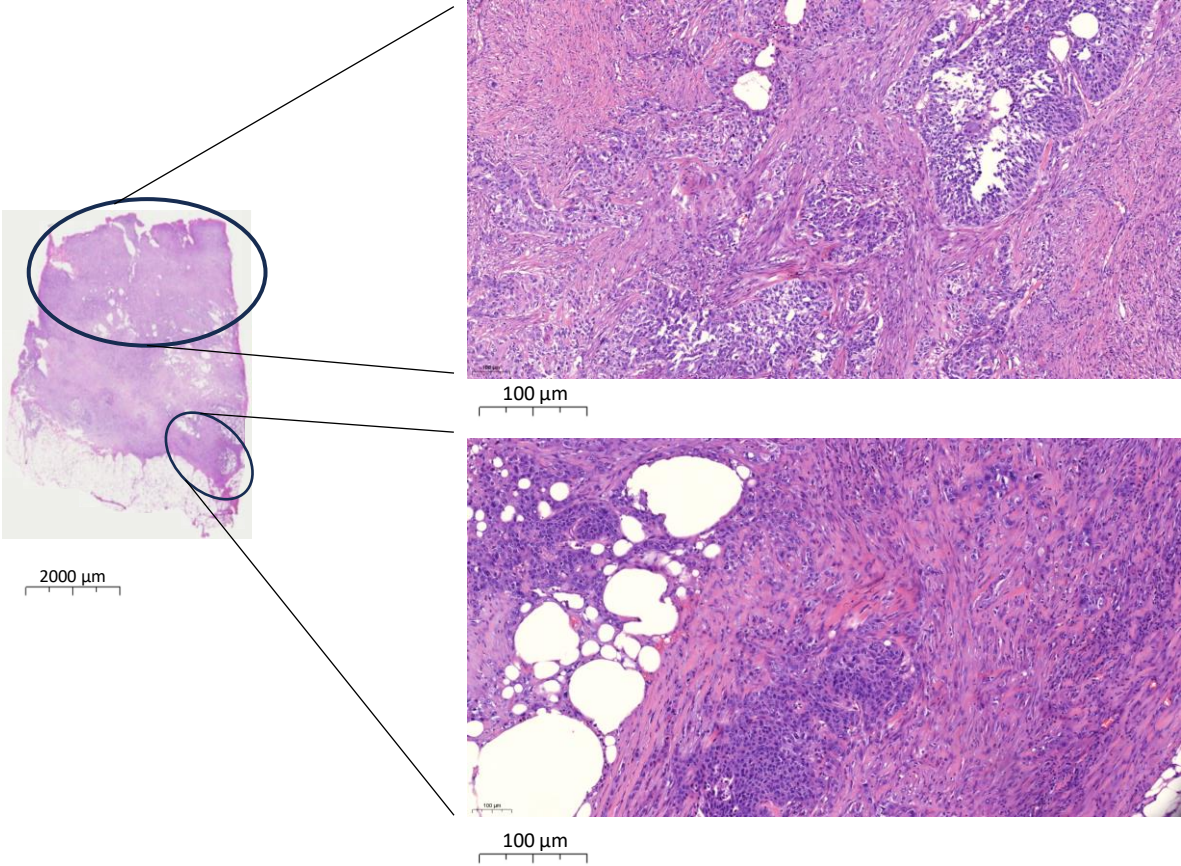

**Tm\_6.2**

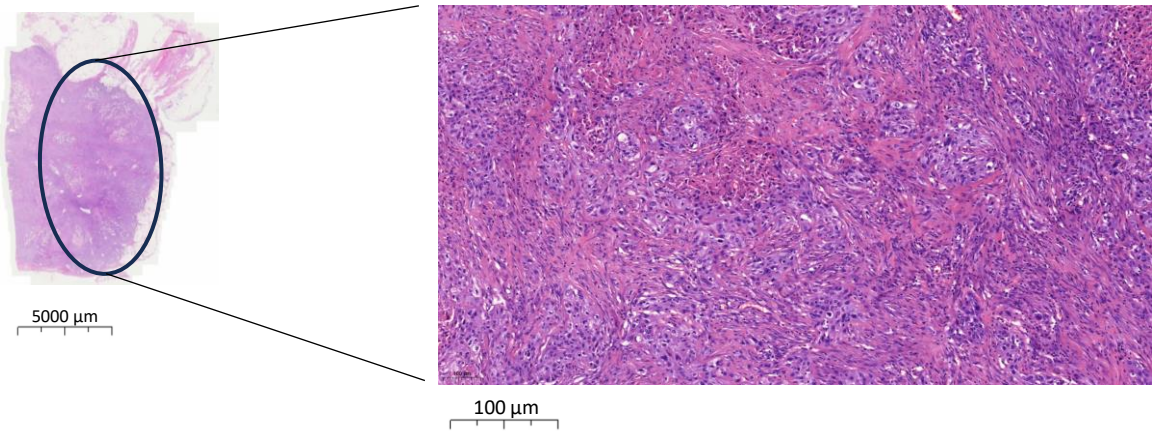

**Tm\_6.3**

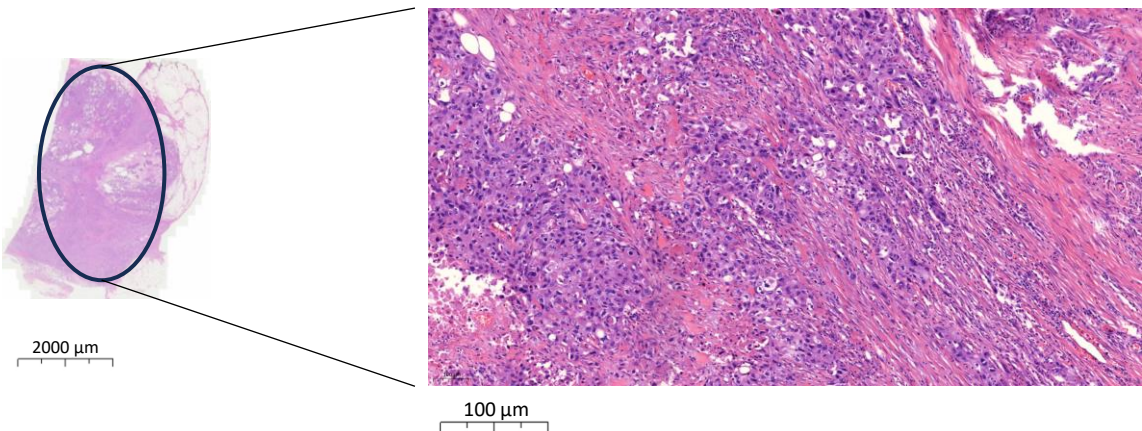

Supplement: Supplementary file 2 [file DataSheet_2.pdf]
